# Effect of volatile compounds produced by the cotton endophytic bacterial strain *Bacillus* sp. T6 against *Verticillium* wilt

**DOI:** 10.1186/s12866-022-02749-x

**Published:** 2023-01-10

**Authors:** Lin Zhang, Yu Wang, Shengwei Lei, Hongxin Zhang, Ziyang Liu, Jianwei Yang, Qiuhong Niu

**Affiliations:** College of Life Science and Agricultural Engineering, Nanyang Normal University, 1638 Wolong Road, Nanyang, 473061 Henan China

**Keywords:** *Verticillium* wilt, Volatiles, Cotton, Biocontrol, *Verticillium dahliae*

## Abstract

**Background:**

*Verticillium* wilt, caused by the fungus *Verticillium dahliae*, leads to significant losses in cotton yield worldwide. Biocontrol management is a promising means of suppressing verticillium wilt. The purpose of the study was to obtain and analyze endophytic bacteria with *Verticillium* wilt-resistant activities from the roots of *Gossypium barbadense* ‘Xinhai15’ and to explore the interactions between the soil and plants.

**Results:**

An endophytic bacterium *Bacillus* sp. T6 was obtained from the *Verticillium* wilt-resistant cotton *G. barbadense* ‘Xinhai15’, which showed significant antagonistic abilities against cotton *Verticillium* wilt. The bioassay results indicated that the strain possessed strong antagonistic abilities that inhibited *V. dahliae* spore germination and mycelial growth without contact, and thus it was speculated that the active factor of the bacteria might be volatile compounds. A total of 46 volatile substances were detected via headspace solid-phase microextraction and gas chromatography–mass spectrometry analysis. The pure product verification experiment confirmed that the styrene produced by the T6 strain was the main virulence factor. Transcriptome analysis showed that following styrene induction, 247 genes in *V. dahliae*, including four hydrolase genes, eight dehydrogenase genes, 11 reductase genes, 17 genes related to transport and transfer were upregulated. Additionally, 72 genes, including two chitinase genes, two protease genes, five transport-related genes, and 33 hypothetical protein genes, were downregulated. The quantitative real-time PCR results confirmed that the expression of the four genes VDAG_02838, VDAG_09554, VDAG_045572, and VDAG_08251 was increased by 3.18, 78.83, 2.71, and 2.92 times, respectively, compared with the uninduced control group.

**Conclusions:**

The research provides a new reference for the development and application of the volatile compounds of endophytic bacteria as new biocontrol agents for the control of *Verticillium* wilt and as biological preservatives for agricultural products.

## Introduction

Cotton (*Gossypium* spp.) is an economically valuable crop species that is cultivated around the world. The most severe fungal disease is vascular wilt, which significantly impacts cotton production globally. The pathogenic fungi of *Verticillium* wilt mainly belong to the genus *Verticillium* [1]. *Verticillium* species that are pathogenic in plants infect a variety of hosts, including valuable agricultural and cash crops [2]. In particular, *Verticillium dahliae* is among most destructive soil-borne pathogens that infects crops. The fungus *V. dahliae* is distributed all over the world and can infect more than 660 species of plants, which causes serious yield losses in crop production. The consequences of *V. dahliae* infection can be far-reaching and global [1]. In particular, it has caused significant economic losses to cotton. The average yield reduction of cotton caused by *Verticillium* wilt is about 10%-35% [3].

The main pathogenic mechanism of *V. dahliae* involves the blocking of blood vessels in the xylem and production of toxins [3]. Alternatively, the mycelium of *V. dahliae* penetrates the plant root surface and colonizes the vascular bundle, causing plant death [4]. Plant pathogens are more difficult to control once they reach the vascular tissue. Methods for controlling disease include the breeding of resistant cultivars, changes in agricultural planting patterns, and chemical control. However, these methods have proven to be less effective and environmentally friendly than expected [5]. Currently, biological control agents provide a promising and environmentally friendly method for the prevention and control of cotton *Verticillium* wilt [6].

Although endophytes are microorganisms that can be isolated from surface-disinfected plant tissues or extracted from plant tissues, endophytes do not harm their host [7]. These microorganisms play numerous important beneficial roles in the metabolism and physiology of their hosts, including atmospheric nitrogen fixation [8], solubilizing phosphates [9], plant growth hormone synthesis [10], toxic compound degradation [11], the inhibition of strong fungal activity [12], and antagonism against bacterial pathogens [13]. Compared to rhizosphere bacteria, endophytes differ in that they are systematically distributed in plants, create a more stable environment, and are reliable and abundant nutrient sources.

Volatile organic compounds (VOCs) are considered potential fungicides because they do not rely on direct contact between antagonistic microorganisms and pathogens [14]. Bacteria produce VOCs with low molecular weights and polarities that can diffuse easily through porous soil structures and across great atmospheric distances. These properties contribute significantly to the potential applications of bacterial VOCs in a variety of environments, including plantations and greenhouses [15].

Recently, several studies on the antifungal activity of microbial VOCs have been carried out in a variety of plant pathogens, such as *Magnaporthe oryzae*, *Fusarium oxysporum*, *Botrytis cinerea* and *V. dahlia* [4]. Altogether, the data indicate that volatile substances have more important functions in microbial interactions compared to non-volatile compounds [16]. In addition, research has shown that the exchange of aerial signals, including VOCs, between microorganisms can alter the metabolism of the recipient microorganisms [17]. This reaction may promote or reduce the production of certain soluble metabolites to ensure the survival of the recipient microorganisms [18]. Compared with larger molecules, VOCs have a greater ability to undergo long-distance diffusion, making them a potential biocontrol agent. Based on this, understanding the specific mode of action of VOC against pathogenic fungi is the key to developing VOCs as new biological fungicides.

The purpose of this study was to explore the endophytes that utilize VOCs to resist cotton *Verticillium* wilt in the roots of the cotton *Gossypium barbadense* ‘Xinhai15’. It was hypothesized that the VOCs produced by the endophytes in verticillium wilt-resistant cotton mediate the interactions between the plant, soil, and pathogens. To test this hypothesis, *Bacillus* sp. T6, an endophytic bacterium that exhibited antagonistic activity against *V. dahliae*, was isolated from the *Verticillium* wilt-resistant cotton variety ‘Xinhai 15’. The VOCs produced by T6 showed antifungal activity in *V. dahlia*. The antagonistic effect of *Bacillus* sp. T6 occurred due to the production of bioactive compounds, including styrene. This study also investigated the molecular mechanism through which styrene inhibited the fungus. The findings of this study provide fresh insights into the mode of action of potential VOC biocontrol agents in addition to providing a reference for the development of environmentally friendly methods of *Verticillium* wilt control.

## Results

### Isolation and identification of strain T6 with antagonistic effects against *V. dahlia* using VOCs

**Fig. 1:**
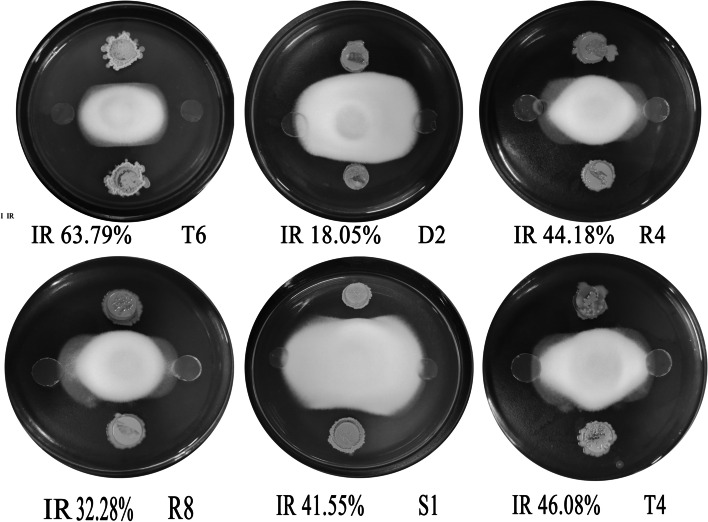
Antifungal activities of the six isolated strains with inhibition activities: The left and right sides of the plate were the culture medium control blocks, and the top and the bottom of the plate were the bacterial blocks, respectively

Thirty-nine endophytic bacterial strains were obtained and isolated from the roots of the *Verticillium* wilt-resistant cotton variety ‘Xinhai 15’. Among them, Proteobacteria (72%) and Firmicutes (23.6%) were the predominant bacterial groups in the ‘Xinhai 15’ root endophytes, particularly the genera *Bacillus* (40.35%), *Enterobacter* (22.97%), and *Pseudomonas* (11.06%). Six bacterial strains exhibited relatively strong fungistatic activity against the pathogenic fungus *V. dahlia* (Fig. 1). These strains accounted for approximately 15% of the endophytic bacteria present in the plants. The inhibition rates of T6 and T4 against *V. dahlia* were 63.79% and 46.08%, respectively, which suggests that among the six endophytic bacteria, the two strains possessed significant inhibitory activity toward the *Verticillium* wilt pathogenic fungus *V. dahlia*.

The six strains were identified according to their morphological properties and the 16S rRNA sequences analysis. These strains consisted of three *Bacillus*, one *Enterobacter*, one *Pseudomonas*, and one *Microbacterium* (Table 1), which suggests that the *Bacillus* genus is the dominant endophyte in the rhizosphere and plays a key role in maintaining cotton plant health.

**Table 1:** Isolation, identification and their VOC inhibition activities of Endophytic Bacteria from XinHai15 roots

Strain	Accessible number	Closest species in 16S rRNA gene sequence database	Similarity (%)	VOC inhibition activity
T4	CP017184	*Enterobacter roggenkampii* EN-117^ T^	100	30.05%
T6	AMXN01000021	*Bacillus subtilis* subsp. *inaquosorum* KCTC 13429^ T^	99.93	95.66%
D2	AB021406	*Pseudomonas beteli* ATCC 19861^ T^	99.44	5.69%
R4	LPVF01000003	*Bacillus halotolerans* ATCC 25096^ T^	99.73	29.85%
S1	Y17227	*Bacillus siamensis* KCTC 13613^ T^	99.86	17.59%
R8	AJVF01000043	*Microbacterium oxydans* DSM 20578^ T^	99.86	11.02%

**Fig. 2:**
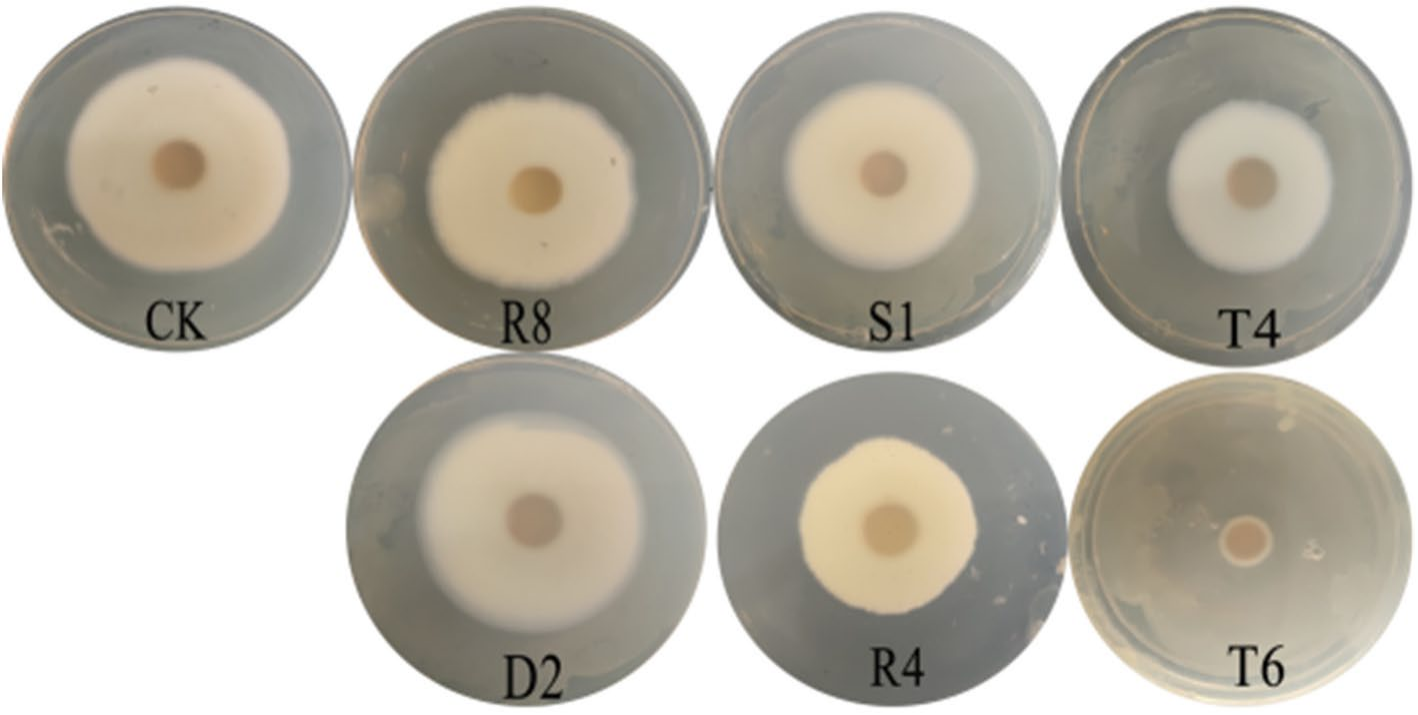
Anti-fungal activity results of volatile substances produced by the six strains tested by inverted plate experiments

The effects of the VOCs of the six strains on antifungal activity were investigated. The GC/MS analysis results on the detection of the VOCs produced by *Bacillus* sp. T6 in 3-day-old cultures are shown in Table 2. The anti-fungal activity results of the volatile substances produced by the six strains were measured by inverted plate experiments. As shown in Fig. 2, the volatile substances produced by the six tested bacteria showed some antibacterial activity. Among them, the inhibition rate of the volatile substances produced by strain T6 reached up to 95.66%, representing the highest inhibition rate. The inhibition rates of the volatile substances produced by the other five bacterial strains were 30.05% (T4), 29.85% (R4), 17.59% (S1), 11.02% (R8), and 5.69% (D2) (Table 1).

**Table 2:** Volatile organic compounds produced by *Bacillus* sp. T6 in 3-day-old cultures detected by GC/MS analysis

RT (min)	RA (%)	Possible compound	Matching degree (%)
2.116	0.29	Octane	86
2.966	15.45	Ethyl Acetate	91
4.028	4.56	Heptane	86
4.202	0.63	Nonane, 3-methyl-	90
5.03	4.47	Decane	95
6.283	0.79	Toluene	90
8.953	1.07	P-Xylene	88
9.543	4.99	Decane, 3,8-dimethyl-	90
10.323	18.60	Dodecane	96
10.579	0.88	3-Undecene, 7-methyl-, (E)-	90
11.134	1.53	Undecane, 3-methylene-	91
11.203	0.32	3-Dodecene, (E)-	91
11.290	2.43	1-Dodecene	96
11.671	1.02	Styrene	98
11.845	0.34	2-Dodecene, (Z)-	96
13.70	4.14	3,5-Dimethyldodecane	88
14.299	11.41	Tetradecane	98
14.485	0.78	Cycloundecane, 1,1,2-trimethyl-	92
14.966	1.16	Tridecane, 3-methylene-	91
15.101	1.25	1-Tetradecene	99
15.564	0.90	2-Tetradecene, (E)-	89
15.942	0.16	7-Tetradecene	89
16.345	0.57	Methoxyacetic acid, 2-tetradecyl ester	87
16.605	0.31	Benzaldehyde	89
17.606	1.95	Hexadecane	96
18.330	0.53	5-Octadecene, (E)-	91
21.053	1.42	Benzene, 1-methoxy-4-(1-propenyl)-	98
25.648	3.05	Caprolactam	99

*RT* Retention time, *RA* Relative peak area (%), the values in that column are the average of two repeats; The VOCs of *Bacillus* sp. T6 with the relative peak areas less than 0.2% and the matching degree less than 85% are not included in this table

The *V. dahliae* did not grow in the soil in which the T6 and R4 strains had been added. However, the fungi did grow well in the soil containing the other four strains, which is similar to that of the control group (Fig. S1), indicating that the T6 and R4 strains could inhibit the growth of *V. dahliae* not only on the Petri dish but also in the soil. Taken together, the T6 strain showed the most significant antifungal activity as a result of its VOCs. Therefore, the T6 strain was selected as the follow-up experimental object.

**Fig. 3:**
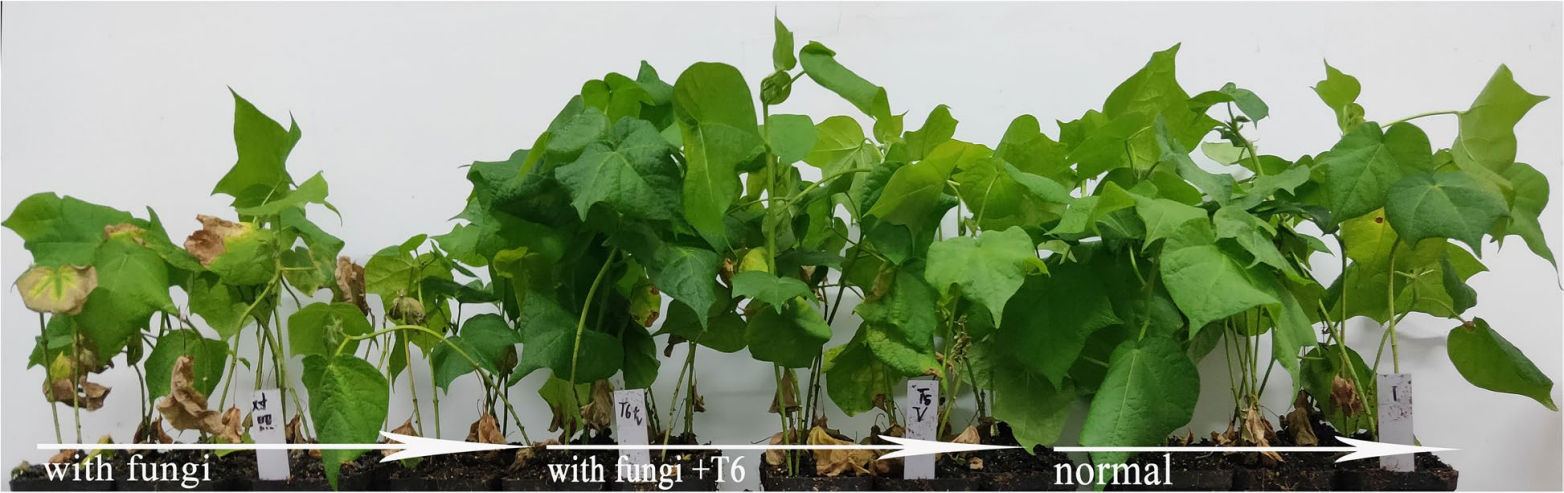
Pot experiments rescreening the strain T6 against *Verticillium* wilt

The results of the pot experiment performed in the light incubator are shown in Fig. 3. The cotton group infected only with the pathogenic fungus suffered from the disease, and the leaves began yellowing and exhibited some defoliation. In addition, infected plants grew more slowly compared to cotton in the blank control group without disease symptoms. However, the cotton irrigated with T6 solution exhibited strong growth and was more resistant to *Verticillium* wilt than the control plants. Under the T6 treatment, the infection rates decreased by 13%-19% compared to the blank control group that was only infected with the fungus, which exhibited a 100% incidence of disease. Repeated experiments revealed that the average control effect of the T6 strain reached 92.55%.

### GC–MS identification of the VOCs of *Bacillus* sp. T6

Based on the HS-SPME-GCMS spectral properties, we identified 28 VOCs in 3-day-old cultures after eliminating the compounds with a matching degree of less than 85% (Table 1). These compounds were classified as alkanes, alkenes, esters, benzenes, acids, and aldehydes. Alkanes and alkenes occupied the vast majority, accounting for 42.9% (12/28) and 35.8% (10/28), respectively.

### Antifungal activity of the selected commercial VOCs

Ten compounds in the VOC profile of *Bacillus* sp. T6 that had a relative content of more than 1% and a matching degree of over 90% were used in the subsequent experiment. The 10 commercially pure compounds were tested for their antifungal activities. Five of the compounds (styrene, 1-tetradecene, 1-dodecene, ethyl acetate, and caprolactam) showed inhibitory activity against *V. dahlia* (Table 3). Of the 10 tested pure compounds, styrene exerted the highest level of antifungal activity and the lowest 50% inhibition concentration values (IC50) for growth inhibition (12.8 μL L^−1^) and conidial germination (7.7 μL L^−1^). Alkanes including decane, dodecane, tetradecane, tridecane, 3-methylene-, and hexadecane showed no detectable inhibitory activity against *V. dahliae*.

**Table 3:** Fifty percent inhibition concentrations (IC50) of ten compounds on the mycelial growth and conidial germination of *V. dahliae*

Compound	CAS number	IC 50, μL L^−1^	
		Mycelial growth	Conidial germination
Ethyl Acetate	000,141–78-6	3699.9	97.2
Decane	000,124–18-5	-^*^	-
Dodecane	000,112–40-3	-	-
1-Dodecene	000,112–41-4	2863.5	96.3
Styrene	000,100–42-5	12.8	7.7
Tetradecane	000,629–59-4	-	-
Tridecane, 3-methylene-	019,780–34-8	-	-
1-Tetradecene	001,120–36-1	2199.6	68.5
Hexadecane	000,544–76-3	-	-
Caprolactam	000,105–60-2	5533.2	49.4

^*^The symbol “-” indicates no inhibitory effect detected

**Fig. 4:**
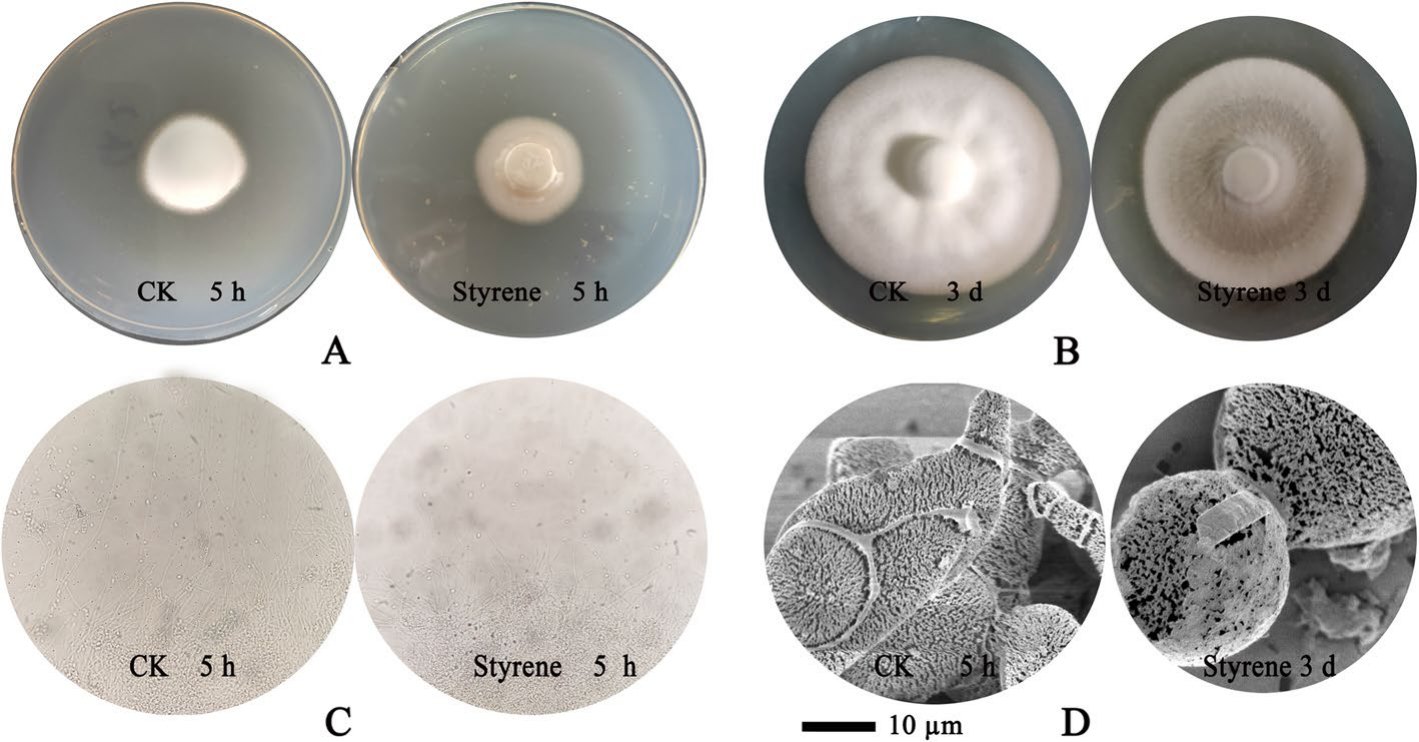
Results of styrene inhibiting fungi *V. dahliae*. **A**. Antifungal activity test of styrene -treated for 5 h on plates; **B**. Antifungal activity test of styrene-treated for 3 d on plates; **C**. Microscopic observation of the fungi after styrene -treated for 5 h; **D**. SEM observation of the fungi after styrene -treated for 3 d

The bioassay experiment results indicated that compared with the control group, the spores of *V. dahliae* hardly germinated when treated with 30 μL of pure styrene for 5 h, and the number of hyphae was significantly reduced (Fig. 4A and C). The inverted plate tests indicated that part of the hyphae of *V. dahliae* was dissolved (Fig. 4B). Moreover, the spore structure appeared incompact and irregular compared with the normal hyphae, and some holes appeared on the surfaces of the spores (Fig. 4D). The hyphae of *V. dahliae* were completely lysed as the treatment duration with styrene was extended to 5 ds.

### Transcriptome analysis of *V. dahlia* in the early response to styrene stress

Six samples were used in transcriptome analysis, consisting of three samples of styrene treatment groups at three different time points (2 h, 4 h, and 6 h) and three corresponding control groups. A high rate of clean reads from each sample was achieved after filtering the raw reads. A total of 46.39 Gb of clean data was obtained, and the clean data of each sample exceeded 7.2 Gb. In total, over 93.92% of the sequences could be mapped to the reference, and all samples had stable GC content with distribution that ranged from 52.70%-55.32%. The Q20 and QC30 values of all samples were 98.29%-98.54% and 94.82%-95.38%, respectively. The results implied successful library construction, and thus the data could be used for subsequent bioinformatics analysis.

An FC > 2 or FC < 0.5, and a q-value ≤ 0.05 were used as thresholds to determine the DEGs. A total of 4818 DEGs (3092 upregulated and 1726 downregulated) were identified between the styrene-treated and control groups. There were 1370 DEGs (952 upregulated and 418 downregulated), 1820 DEGs (909 upregulated and 911 downregulated), and 1628 DEGs (1231 upregulated and 397 downregulated) at the three time points of styrene induction for 2 h, 4 h, and 6 h, respectively. The Venn diagram showed that there were 319 DEGs including 247 upregulated and 72 downregulated genes that were common among the three groups. The DEGs were further analyzed using GO enrichment and KEGG analyses. In detail, the genes that were upregulated in the styrene treatment compared to the control were significantly enriched in the biological process, molecular function, and cellular component categories, in addition to being associated with metabolic enzymes, stress-stimulated response proteins, regulation factors, and membrane component proteins. The downregulated genes in the styrene treatment versus the control were mostly involved in the transport and catabolism, cell growth, and biosynthesis categories, specifically peptidase, lipase, proteases, chitinases, and methionyl-tRNA synthetase.

### Expression levels of genes related to growth and apoptosis

**Fig. 5:**
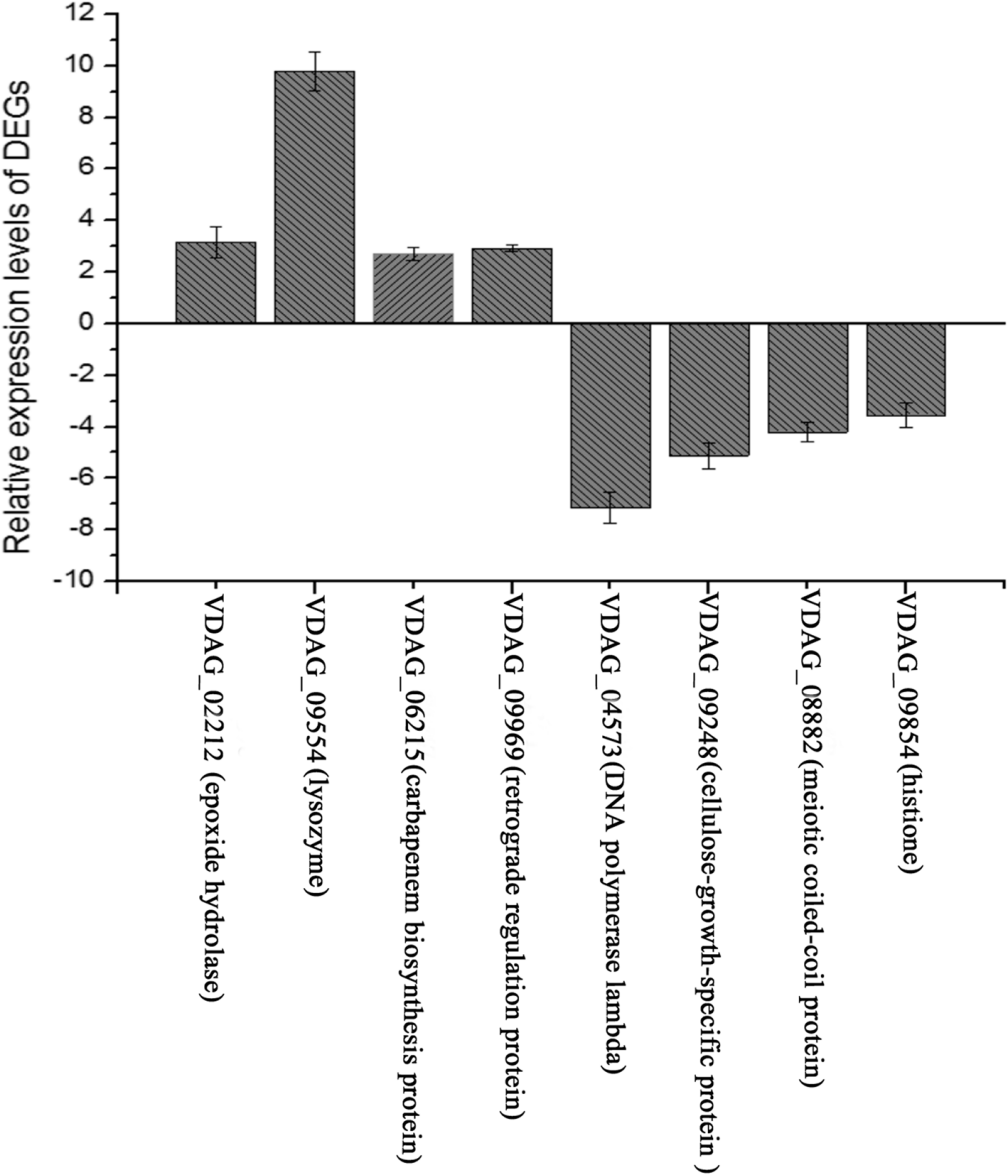
Expression levels of genes related to growth by RT-qPCR. The x-axis indicates the gene names. The left y-axis indicated relative expression level of RT-qPCR. Error bars represent standard error of mean

Transcriptome sequencing technology can be used to obtain a large volume of data on the DEGs involved in specific biological processes. The eight genes concerned with metabolic process and response to stimulus were screened from the DEGs based on the differential expression levels. The expression levels of genes related to growth and stress (VDAG02212, VDAG06215, VDAG09554, and VDAG09969) were upregulated, while VDAG04573, VDAG08882, VDAG09248, and VDAG09854 were downregulated in the styrene-treated group compared to the untreated control fungi. In addition, the qRT-PCR results indicated that the expression patterns of the eight genes were identical to those detected by transcriptome sequencing (Fig. 5). The relative expression of genes related to lysozyme, epoxide hydrolase, retrograde regulation protein, and carbapenem antibiotics biosynthesis protein was upregulated by 9.78, 3.18, 2.92, and 2.71 times, respectively, compared with the control group. The relative expression of genes related to DNA polymerase lambda, meiotic coiled-coil protein, cellulose-growth-specific protein, and histone was downregulated by 7.15, 5.11, 4.20, and 3.55 times compared with the control group, respectively. The findings verified the reliability of the RNA-seq data and further indicated that styrene was the virulence factor in the T6 strain that inhibited the *V. dahliae* growth.

## Discussion

The study of the interactions between microbiota and their host plants has become increasingly important. The microbiome associated with plant roots represents a vast biodiversity reservoir containing tens of thousands of species [18]. The beneficial interactions between roots and microbes are necessary for plants to absorb nutrients and increase their pathogen resistance tolerance to various stresses [16–20]. Microbial composition has been identified in a variety of plant species, such as *Arabidopsis* [21–23], *Populus* [24, 25], maize [26], rice [26, 27], and cotton [28, 29].

Cotton is a valuable cash crop cultivated around the world. *Verticillium* wilt caused by the soil-borne fungal pathogen *V. dahliae* is the most destructive disease in cotton. *Verticillium* wilt resistance is mediated by quantitative trait loci, and these quantitative characteristics may be significantly affected by other factors, including environmental factors and plant-associated microbiota. The elementary inocula of *V. dahliae* consist of microsclerotia-fungal static structures in dead plant tissues and soil. Microsclerotia may survive in the soil for more than 10 years in the absence of a host [28]. Various cultivars differ in their susceptibility to *V. dahliae* [30]. Wei et al. [29] have reported that specific rhizosphere and endosphere microbes may greatly improve *V. dahliae* resistance in cotton. In addition, several well-known taxonomic groups that exhibit *Verticillium* wilt resistance contained potentially profitable microbes, including *Pseudomonadales*, *Bacillales*, *Trichoderma* and *Rhizobiales*, with higher relative abundances found in resistant cultivars. Data obtained in greenhouse research supported the action of beneficial rhizosphere microbes in reducing the development of *Verticillium* wilt. The tolerance on the *Verticillium* wilt has been reported to be closely involved in well-known beneficial bacteria, including *Bacillus* [31], *Lysobacter* [32], *Streptomyces* [33], *Rhizobiales* [29], and *Pseudomonas* [34]*.* The relative abundances found in wilt-susceptible cultivars, including the fungal endophytes *Aspergillus aculeatus*, *Alternaria solani*, *V. longisporum* and *Choanephora* as well as the rhizosphere fungi *Magnaporthe grisea*, Ustilaginaceae, Ceratobasidiaceae, *Thielaviopsis basicola* and *Alternaria,* were increased in many fungal groups [28, 35].

In addition to numerous effective mechanisms of cooperation that can act synergistically to combat phytopathogens [36], *Bacillus* spp. are able to form endospores to promote their long-term maintenance and survival under a variety of environments. The use of these VOCs in disease control is advantageous, and VOCs are ideal informational chemicals due to their ability to function over long distances through the air and diffuse via soil pores [37]. The VOCs of the bacteria *Bacillus* genus exhibit potential antagonistic behavior against several phytopathogens. It has been reported that VOCs produced by *B. subtilis* and *B. amyloliquefaciens* exhibit up to 87% inhibition against the fungus *Rhizoctonia solani*. These compounds lead to the deformation and death of fungal hyphae, which has been verified via scanning electron microscopy observations [38]. Other researchers have also detected hyphal and spore deformations in the phytopathogenic fungus *Macrophomina phaseolina* caused by volatile compounds produced by *B. subtilis* and *B. amyloliquefaciens* [39, 40]. Moreover, volatile compounds from *Bacillus* spp. ACB-65 and *Bacillus* spp. ACB-73 provided 86% inhibition in the control of *Phyllosticta citricarpa* [41]. In addition, Gotor-Vila et al. (2017) verified the use of *B. amyloliquefaciens* VOCs in the control of *Monilinia laxa*, *Monilinia fructicola*, and *B. cinerea* in post-harvest sweet cherry fruits, thereby reducing the incidence of disease and sporulation of pathogens [39]. However, little is known about the application of *Bacillus* VOCs in plant disease control, as well as the chemical composition of *Bacillus* VOCs [4].

In this study, we investigated the *Bacillus* sp. T6 strain from the roots of the endophytic bacteria in *Verticillium* wilt-resistant cotton, which showed strong inhibitory activities against *V. dahliae* via its VOCs. The GC–MS results and pure product verification experiments showed that the styrene produced by the T6 strain represented an important antagonistic virulence factor. In the fungi treated with styrene treatment, the hyphae were obviously reduced and fell off, and the morphology of the spores was distorted and deformed. The antifungal mechanism of styrene was further explored at the molecular level using transcriptome and RT-qPCR analysis. Highly expressed genes in the styrene-treated samples included VDAG-09554 (lysozyme), VDAG-02212 (epoxide hydrolase), VDAG-06215 (carbapenem biosynthesis protein), VDAG-09969 (retrograde regulation protein), VDAG-02980 (cell wall protein PhiA), VDAG-07280 (LEA domain-containing protein), VDAG-07553 (NAD dependent epimerase/dehydratase family protein), VDAG-00511 (glucan 1,3-beta-glucosidase), and VDAG-05117 (surface protein). Genes with low expression following styrene treatment included VDAG-04573 (DNA polymerase lambda), VDAG-08882 (meiotic coiled-coil protein), VDAG-09248 (cellulose-growth), VDAG-09854 (histone), VDAG-02726 (ribosome biogenesis protein Sqt1), VDAG-08736 (peptidoglycan binding domain-containing protein), and VDAG-05404 (polysaccharide biosynthesis protein vipA/tviB). Analysis of the expression levels of the eight candidates was performed in *V. dahliae* in response to styrene. The most significantly up-regulated gene was VDAG-09554, which is a lysozyme gene. Lysozyme is a muramidase that cleaves peptidoglycan, targeting the glycosidic bond between β-1,4-linked residues of N-acetylmuramic acid (NAM) and N-acetylglucosamine (NAG) [42]. In addition, lysozyme is an innate immune system component that causes cell lysis [43]. We inferred that styrene strongly induced lysozyme production in *V. dahlia*, resulting in fungal apoptosis. The most significantly down-regulated gene was VDAG-04573, which is a DNA polymerase gene. DNA polymerases are critical to the survival of all life forms and are the only enzymes with the ability to duplicate the genetic information stored in nucleic acid DNA. DNA contains the genetic information for all organisms, which therefore require at least one DNA polymerase to ensure their survival. It can be inferred that styrene inhibits the growth of the fungi via preventing the expression of DNA polymerase. The results confirmed that the expression levels of the genes related to cell lysis and transportation in *V. dahliae* were upregulated, while the expression of growth-related genes was downregulated after styrene induction, thus inhibiting the growth of the fungus *V. dahliae*.

At present, VOCs obtained from antifungal bacteria, filamentous fungi, and yeast are used to control pathogenic fungi affecting fruits and vegetables [14]. Nagrale et al. reported three cotton endophytic rhizospheric bacteria, including *B. cereus* CICR-D3, *B. aryabhattai* CICR-D5, and *B. tequilensis* CICR-H3, which produce mVOCs and have antagonistic potential against the fungal pathogen *Macrophomina phaseolina* [44]. The major antifungal mVOCs produced by these strains include benzene, 1, 3-diethyl- and benzene, 1, 4-diethyl, naphthalene, m-ethylacetophenone, and ethenone [44]. It is thus evident that the antifungal VOCs from cotton rhizo- and endophytic bacteria have potential application prospects in the fungal disease management of cotton. The successful commercial application of VOCs depends on a thorough understanding of the antifungal mechanisms of VOCs. However, limited information exists regarding the molecular and physiological mechanisms by which VOCs control fungal diseases. Some studies have shown that microbial VOCs can damage cell walls and membrane structure, resulting in intracellular lysate leakage and oxidative stress induction [14, 20, 45–47].

The present work demonstrated that the volatiles produced by the bacterial isolates could be used for the sustainable management of cotton *Verticillium* wilt caused by *V. dahlia*. Ethylene produced by the cotton endophytic bacterial strain *Bacillus* sp. T6 in the *Verticillium* wilt-resistant cotton *G. barbadense* ‘Xinhai15’ radically promoted the expression of the lysozyme gene in the fungi, which causes the autolysis of fungal cells. In addition, ethylene triggers the accumulation of ROS as well as oxidative stress in fungal cells. It can be inferred that the disruption of redox homeostasis triggered harmful ROS accumulation, leading to cell dysfunction and death. The results provide a promising approach for managing cotton *Verticillium* wilt and enriching the resources of VOC-producing antagonists. Further in-depth studies are necessary to thoroughly elucidate the antifungal mechanisms of VOCs to develop antifungal bacteria that can be used in commercial applications.

## Conclusions

Herein, we found that an endophytic bacterium, *Bacillus* sp. T6, isolated from the *Verticillium* wilt-resistant cotton 'Xinhai15' possessed strong inhibition against spore germination and mycelial growth without contact. Further, we found that the styrene produced by the T6 strain was the main virulence factor. The molecular mechanisms of the interactions between the volatile compound styrene and cotton plants were explored by transcriptome analysis and RT-qPCR. The results revealed the mode of action of *Bacillus* sp. T6 as a bio-control agent against *Verticillium* wilt.

## Methods

### Microbial strains and materials

The fungus *V. dahliae* V991, the *Verticillium* wilt-resistant cotton ‘Xinhai 15’, and *Verticillium* and the sensitive strain TM-1 were obtained from the State Key Laboratory of Cotton Biology at Henan University. The antagonistic strain Bacillus sp. T6 was screened and obtained from the roots of *Verticillium* wilt-resistant cotton ‘Xinhai 9’ samples, and the strain was deposited in the China Center for Type Culture Collection (CCTCC M2019618). The fungi were grown on Potato Dextrose Agar (PDA) plates (20.0% potato, 2.0% glucose, and 1.5% agar). The bacterial strains were incubated on Luria–Bertani culture media (1.0% tryptone, 0.5% yeast extract, and 0.5% NaCl). All of the chemical reagents used in this study were purchased from Sigma (St. Louis, MO, USA).

### Screening and identification of the biocontrol strains against *V. dahlia*

The experiments on the screening and identification of the biocontrol strains against *V. dahlia* were performed based on a previous article [27]. In brief, nine samples of ‘Xinhai 15’ roots were collected, surface-disinfected with 75% ethanol for 30 s followed by 1 L of 0.1% mercury for 7 min, frozen with liquid nitrogen, and ground for 15 min using a 1-mL micro-dismembrator (Wheaton). Then, the sterilized solution obtained following the final rinse was cultured on nutrient and oligotrophic agar plates to verify whether successful surface sterilization had occurred (0 cfu indicated successful sterilization). After serial dilution with sterile water, approximately 50 μL of the suspension was plated onto LB and incubated at 28 °C for 3 d to culture the endophytic bacteria. The bacteria were selected based on the differences in the color, gloss, shape, and size of the colonies and were purified by streaking them onto the LB solid medium using an inoculating loop at least three times.

The antifungal activities of the obtained endophytic bacteria were tested via a dual culture method [48]. The fungus *V. dahliae* V991 was cultivated for 7 ds at 25 °C on PDA medium. Then, 200 µL aliquots of the cultured liquid bacterial suspensions were spread onto LB agar plates and incubated for 12 h at 37 °C. A fungal block 1 cm in diameter was fixed at the center of the PDA plate. Equivalent amounts of tested bacterial strains and blank LB block (0.5 cm × 0.5 cm × 0.3 cm) were placed on both sides of the same plate. All isolates were incubated for 3–5 d at 25 °C, and the fungal mycelial growth was observed. Finally, the inhibition rate (IR) was calculated using the following equation: (control colony diameter − processing colony diameter) × 100% / (control colony diameter − fungus cake diameter). All tests were repeated in triplicate.

To further evaluate the antagonistic activities of the bacterial strains, a pot experiment was performed using the previously reported method. In the experiment, the *V. dahliae* V991 spores were added to the soil (loess:black soil:vermiculite 1:1:1) and mixed to obtain a concentration of 1 × 10^6^ spores/g of soil. After combining the soil and fungal spores, 30 seeds disinfected in advance were immediately sown into the soil mixture. Following that, the bacterial liquid to be tested was cultured and adjusted to OD_600 nm_ = 1.0 using sterile water. Then, 10 mL of the test bacterial suspension (OD_600 nm_ = 1.0) + 90 mL of sterile water was added into the spore soil as the test group, while 100 mL of sterile water constituted the blank group. Ten milliliters of T6 cultural solution per pot of cotton was added every 5 d. The cottonseeds (TM-1) were planted in the soil and grown at 25 °C in a light incubator with a 16/8 h light/dark photoperiod. Following 30 d of incubation, the disease indices (DIs) were counted every 5 d. The DI was based on the following criteria: healthy plants = 0; less than one or part of the cotyledons turned yellow or necrotic = 1; two cotyledons turned yellow or necrotic = 2; one true leaf turned yellow or necrotic = 3; two or more true leaves yellowing or necrosis = 4. DI = ∑ (number of stages × number of plants) / (highest level value × total number of plants) × 100%. Each pot trial was repeated three times in triplicate.

The pure isolate strains of the endophytic bacteria that exhibited the strongest antagonism against *V. dahlia* were identified based on morphological examination and 16 s rRNA gene sequence analysis.

### Exploration of the inhibition factor and gas chromatography (GC)-mass spectrometry

The thermostability of the antifungal metabolites of the bacteria was assessed after boiling for 10 min. In the first step, 40 μL of the 1.0 × 10^6^ CFU/mL *V. dahliae* spore suspension was spread on a PDA plate. Then, 200 μL of boiled sterile fermentation filtrate and the untreated control were separately placed into holes at equal distances (2 cm) from the center of the plate. The suspension was then incubated at 25 °C for 5 d. The germination of the fungal spores and the diameter of the inhibition zone were measured. The test was repeated three times.

In vitro assays of the VOCs produced by the endophytic bacteria were evaluated against the mycelial growth of the pathogenic fungi as follows. The ‘upside-down plate’ method was used with PDA culture medium for fungi above and LB for bacteria below. First, *V. dahliae* was cultured in PDA medium for 14 ds, and a 1-cm-diameter hole punch of the cultured fungi clump was obtained and placed in the center of the PDA medium for incubation at 25 °C for 3 ds. Then, 100 μL of the screened bacterial liquid was spread evenly on fresh LB solid medium and incubated at 37 °C for 12 h. Finally, the pre-raised *V. dahliae* plate was inverted and placed inside the bacteria dish. The *V. dahliae* group was inverted on the blank LB solid medium for use as a negative control. The reversed plate was incubated at 25 °C for 15 ds, and the IR was calculated with the following equation: (control colony diameter − processing colony diameter) 100% / control colony diameter. All tests were repeated in triplicate [49].

The chemical components of the “VOC” of the T6 bacteria were analyzed with gas chromatography-mass spectrometry (GC–MS). The VOCs in the cultured fermentation broth of the strain were collected using the headspace solid-phase microextraction technique (HS-SPME) (Wan MG 2008). The VOC components were identified using a GC–MS machine (6890 N-5975B, Agilent Technologies Inc., CA, USA) following the procedures recommended by the manufacturer. Eight synthetic chemicals (1-dodecene, styrene, tetradecane, hexadecane, ethyl acetate, decane, caprolactam, and 1-tetradecene) were selected with reference to the VOC profile of T6. Only these compounds were available for purchase from chemical companies. The commercial pure products were purchased from Sigma-Aldrich Company. Using the dual culture method described above, the chemicals were tested individually to determine their ability to inhibit the mycelial growth and conidial germination of *V. dahliae*. The concentration value for 50% inhibition of mycelial growth and conidial germination (IC50), expressed as microliters per liter (μL L^−1^), was inferred based on the data on the inhibition percentages and the corresponding VOC doses used in the double-dish assay (Toral et al. 2021). Moreover, the morphological characteristics of the hyphae of *V. dahliae* treated with pure VOC were observed using scanning electron microscopy to assess the VOC suppression mechanisms.

### Transcriptome analysis of *V. dahliae* in response to styrene

IT was found that the styrene produced by T6 showed the maximum antifungal activity through antagonistic growth assay. The *V. dahliae* was cultured in Czapek-Dox broth medium for 3 d, and then 60 mL of the cultured fungal liquid was extracted and evenly distributed into three 150 mL conical flasks. The sterilized 10 mL Eppendorf tube was placed into a conical flask with the mouth facing upward. One hundred microliters of 10^−6^ styrene were added into the Eppendorf tube. The fungi with styrene were cultured in a shaker at 200 rpm and 25 °C for 2 h, 2.5 h, and 3 h. Fungi without any styrene added were used as a negative control. The four groups of samples were collected quickly and placed in liquid nitrogen to be frozen.

The total RNA was isolated from fungal samples with a Fungal RNA Kit (OMEGA, Beijing, China). The RNA quality was measured using an Agilent 2100 Bioanalyzer with an RNA 6000 Nano Kit (Agilent Technologies, Beijing, China). The Ribo-Zero AQ8 Kit was used to remove ribosomal RNA. Equal amounts of RNA obtained from each sample were used in the construction of the cDNA library with the NEB Next Ultra Directional RNA Library Prep Kit for Illumina (NEB, Ipswich, MA, USA) following the manufacturer’s instructions. The cDNA fragments were purified with a QiaQuick PCR extraction kit and end-repaired. Following the addition of poly (A), all fragments were ligated to Illumina sequencing adapters. The ligation products were size-selected via agarose gel electrophoresis, amplified using PCR, and sequenced in the Illumina HiSeqTM 4000 System with the 2 150 bp paired-end read module produced by Gene Denovo Biotechnology Co. (Guangzhou, China). Transcriptome assembly and characterization and differential gene expression analysis were performed following standard procedures.

The edgeR package (version: 3.10.2) with default parameters was used to conduct differential gene expression analysis. Transcripts with a fold change ≥ 2 and a false discovery rate (FDR) < 0.05 were considered to be significantly differentially expressed genes (DEGs). Gene enrichment analysis was performed on the DEGs obtained from each sample to determine their Gene Ontology enrichment categories with BLAST2GO (version: 2.3.5). Significant terms were set at FDR < 0.05. Based on the Kyoto Encyclopedia of Genes and Genomes (KEGG) unigene database annotation, the DEGs in each comparison were analyzed to determine pathway enrichment, and pathways with FDR < 0.05 were considered significantly enriched.

### Molecular docking of styrene

The transcriptome analysis results showed that proteins with significant differences in the gene expression levels after treatment styrene with were used in docking simulations. Three-dimensional structures of these proteins were downloaded from the Protein Data Bank (PDB) and optimized with Discovery Studio. After the bound water, hydrogen atoms, and other unneeded molecules were removed, polar hydrogen atoms were added. The styrene file was generated using Visualizer Studio 3.1 molecular docking to explore the interactions between styrene and proteins with AutoDock 4.2 (Autodock Molecular Graphics Laboratory, the Scripps Research Institute, La Jolla, CA, USA) (Morris et al. 2009). The parameters employed for molecular docking included a Lamarckian genetic algorithm (LGA) with a population of 100 individuals, a maximum of 2,500,000 energy evaluations, and a maximum of 27,000 generations, while the remaining parameters were set to the default values.

### Quantitative real-time (qRT) PCR validation of differential gene expression

Combined with gene differential expression analysis and functional annotation, the eight key genes in response to the growth inhibition of the fungus *V. dahliae* were selected and verified by RT-qPCR. An RNAclean Kit (BioTeck, China) was performed to purify the total RNA following total RNA isolation. The RNA concentration was obtained by determining the absorbance at 260 nm with a UV spectrophotometer. Following the generation of random-primed cDNAs, RT-qPCR analysis was conducted using a SYBR Green JumpStart Taq Ready Mix for qPCR kit (Sigma-Aldrich Co) according to the manufacturer’s instructions. The partial 18S rRNA sequence amplified by primers N1 (ACGAGATCAGGACGGGCTT) and N2 (CGGCGTCTTCTGGAACATTTC) was used as the internal control. The primers of the eight tested genes were designed, and their sequences are shown in Fig. 1. The PCR amplification consisted of 40 cycles of 94 °C for 30 s, 60 °C for 31 s, and 72 °C for 40 s on an ABI PRISM 7000 Real-Time PCR machine.

## Availability of data and materials

The transcriptome sequence data generated and analyzed in this work have been submitted to the NCBI (https://www.ncbi.nlm.nih.gov/) and are available under the following accession number: BioProject PRJNA912315.

## Data Availability

All data and material are available upon request to the corresponding author.

## Abbreviations

VOCs: Volatile organic compounds
SEM: Scanning electron microscope
RT-qPCR: Real-time quantitative PCR
